# Hepatocyte Growth Factor Mediates the Antifibrogenic Action of *Ocimum bacilicum* Essential Oil against CCl_4_-Induced Liver Fibrosis in Rats

**DOI:** 10.3390/molecules200813518

**Published:** 2015-07-23

**Authors:** Hanan A. Ogaly, Nadia A. Eltablawy, Adel M. El-Behairy, Hatim El-Hindi, Reham M. Abd-Elsalam

**Affiliations:** 1 Department of Biochemistry and Chemistry of Nutrition, Faculty of Veterinary Medicine, Cairo University, Giza 12211, Egypt; E-Mails: adelelbehairy@gmail.com (A.M.E.-B.); hatimelhindi@gmail.com (H.E.-H.); 2 Biochemistry Division, National Organization for Drug Control and Research (NODCAR), Giza 12111, Egypt; E-Mail: nadia_eltablawy@yahoo.com; 3 Department of Pathology, Faculty of Veterinary Medicine, Cairo University, Giza 12211, Egypt; E-Mail: reham_pathology@hotmail.com

**Keywords:** *Ocimum basilicum*, liver fibrosis, antioxidant, α-SMA, CYP2E1, HGF

## Abstract

The current investigation aimed to evaluate the antifibrogenic potential of *Ocimum basilicum* essential oil (OBE) and further to explore some of its underlying mechanisms. Three groups of rats were used: group I (control), group II (CCl_4_ model) and group III (OBE-treated) received CCl_4_ and OBE 2 weeks after the start of CCl_4_ administration. Oxidative damage was assessed by the measurement of MDA, NO, SOD, CAT, GSH and total antioxidant capacity (TAC). Liver fibrosis was assessed histopathologically by Masson’s trichrome staining and α-smooth muscle actin (α-SMA) immunostaining. Expression of hepatocyte growth factor (HGF) and cytochrome P450 (CYP2EI isoform) was estimated using real-time PCR and immunohistochemistry. OBE successfully attenuated liver injury, as shown by histopathology, decreased serum transaminases and improved oxidative status of the liver. Reduced collagen deposition and α-SMA immuopositive cells indicated an abrogation of hepatic stellate cell activation by OBE. Furthermore, OBE was highly effective in stimulating HGF mRNA and protein expression and inhibiting CCl_4_-induced CYP2E1 down-regulation. The mechanism of antifibrogenic action of OBE is hypothesized to proceed via scavenging free radicals and activating liver regeneration by induction of HGF. These data suggest the use of OBE as a complementary treatment in liver fibrosis.

## 1. Introduction

Liver fibrosis and its end stage consequence, cirrhosis, remain as the major causes of morbidity and mortality worldwide with increasing social and economic impacts [1]. Despite the tremendous advances in the field of modern medicine, efficient and well-tolerated anti-fibrotic drugs are yet to be developed. The current treatment of hepatic fibrosis is limited to the withdrawal of the noxious agents and orthotropic liver transplantation in the late stages [2,3]. Thus, the search for new medicines is still ongoing.

Studies over the past decade have focused on the mechanism of fibrosis and fibrogenic cells that generate the scarring response known as hepatic stellate cells (HSCs). In response to persistent chronic inflammation, HSCs are activated and transformed into myofibroblast-like cells which then proliferate and produce an extracellular matrix (ECM) [4]. Oxidative stress, the result of the imbalance between production and clearance of reactive oxidative species (ROS), represents a common feature in the different types of liver injuries [1]. *In* *vitro* and *in vivo* data have suggested the involvement of ROS in the pathogenesis of fibrosis [1,3]. ROS together with inflammatory cytokines and growth factors induce HSC activation. Therefore, any intervention aimed at reducing the exposure of HSCs to these oxidative and inflammatory stimuli could slow down or inhibit the progression of fibrosis.

Herbal-based therapeutics have garnered a lot of attention as a promising approach for the treatment of and prophylaxis against fibrosis [5]. Among the herbal products, essential oils are increasingly being referred to as rich sources of bioactive constituents, *i.e.*, monoterpenes, sesquiterpenes, and phenylpropanoids. Many studies have shown that these chemical classes have several biological activities [6]. *Ocimum basilicum* (OB., sweet basil, or *Rehan* in Egypt) is an annual, widely cultivated herb known for its medicinal value in both traditional medicine and modern pharmacological investigations. Most of the biological activities reported for sweet basil like antioxidant, anticancer, and antibacterial activities are associated with its volatile oil content [6,7]. Owing to the potent radical scavenging and antioxidant activity of basil, its aqueous extract has been found to possess potential hepatoprotective and antifibrotic effects in different experimental models of liver toxicity and fibrosis [7,8]. Although, several terpenoid constituents of basil oil are known as hepatoprotective agents [9], the antifibrogenic activity of the essential oil of this plant has never been evaluated.

In the present study, we sought to investigate the efficacy of OBE to improve the liver function and oxidative status and prevent the progression of liver fibrosis. We provide evidence to support the notion that the antifibrogenic effects of OBE are likely mediated by upregulation of HGF.

## 2. Results and Discussion

### 2.1. Chemical Composition of OBE

Qualitative and quantitative analysis of hydrodistilled OBE using gas chromatography coupled with mass spectrophotometry (GC-MS) revealed the presence of fifteen chemical constituents, representing about 99.6% of the total composition. In Table 1 the components are listed in the order of their elution on the HP-5872 column. OBE was predominantly composed of monoterpene hydrocarbons (70.3%), mainly the oxygenated monoterpene *iso*-menthone (38%), and lesser amounts of 1.8-cineole (13.9%), *trans*-sabiene (12%), and pulegone (6.4%). We also noted the presence of methyl eugenol (7.5%), which is a phenylpropanoid, as a constituent of OBE. Small amounts of terpenoids (L-carvone) and sesquiterpenes (*cis*-α-bisabolene, *trans*-α-bergamotene, α-farnesene, α-humulene, *trans*-caryophyllene δ-cadinene and α-morphene) were also found in OBE.

**Table 1:** Chemical composition of OBE by GC/MS.

Retention Time (Rt) (min)	Components	Relative %
5.1	1,8-Cineole	13.90
7.1	*trans*-Sabinene	12.00
8.2	*iso*-Menthone	38.00
8.9	Pulegone	6.40
9.1	L-Carvone	3.50
9.4	Methyl acetate	1.80
10.9	Methyl eugenol	7.50
11.1	*trans*-α-Bergamotene	3.60
11.2	α-Farnesene	4.10
11.4	α-Humulene	0.90
12.1	α-Amorphene	2.00
12.3	*cis*-α-Bisabolene	1.30
12.8	*trans*-Caryophyllene	0.80
13.5	δ Cadinene	0.90
22.1	Dioctyl phthalate	2.90
	Total identified	99.60

### 2.2. Effect of OBE on Liver Functions in CCl_4_-Treated Rats

CCl_4_ administration significantly increased the serum levels of liver transaminases (ALT and AST) over the normal group (Table 2). OBE treatment at a dose of 200 mg·kg^−1^ showed a high capacity to lower the increased levels of these enzymes.

**Table 2:** Effect of OBE on serum ALT and AST in CCl_4_-induced liver fibrosis.

Parameter	Group I	Group II	Group III
ALT (U/L)	19 ± 0.45 ^a^	1111.2 ± 34.5 ^c^	349.2 ± 16.70 ^b^
AST (U/L)	41 ± 1.70 ^a^	1005.8 ± 47.56 ^c^	313.8 ± 24.05 ^b^

Group I: control; group II: CCl_4_; group III: OBE-treated group. Values are mean ± SE (*n* = 5). The presence of different superscripts means significant differences between groups in the same row. ANOVA test followed by Duncanʼs multiple comparisons between groups at *p* < 0.05 were employed.

### 2.3. Antioxidant Effect of OBE against CCl_4_-Induced Oxidative Liver Damage

The obtained results proved that CCl_4_ was able to induce oxidative liver injury. The CCl_4_ treated group (II) showed a statistically significant elevation in the level of MDA, a late biomarker of oxidative stress and a good indicator of the degree of LPO, to 169% as compared to control group. Also, a significant increase in NO concentration (310%) was observed in the fibrotic group (Table 3). On the other hand, Group II showed severe reduction in the activity of the antioxidant enzymes SOD and CAT with a dramatic depletion in GSH levels to 30.2%, 45.3% and 22.6% of the control levels, respectively (Table 4). In the same line, a significant reduction in TAC to 47.7% was obtained. Rats receiving OBE concurrently with CCl_4_ for 6 weeks (group III) showed significantly suppressed MDA and NO levels compared to group II (Table 3). Moreover, the dramatic depletion of GSH by CCl_4_ was reversed and increased to 58.5% of the control level. Furthermore, OBE improved SOD and CAT activities to 81.9% and 74.2%, respectively (Table 4), whereas a significant elevation of TAC was clear in the group III, reaching 81.8% of the control level (Table 4).

**Table 3:** Effects of OBE treatment on oxidative stress biomarkers in CCl_4_ fibrotic liver.

Parameter	Group I	Group II	Group III
**MDA (µM/g liver)**	66.8 ± 2.5 ^a^	112.62 ± 3.4 ^c^	86.3 ± 3.6 ^b^
**NO (mmol/L)**	4.47 ± 0.27 ^a^	14.11 ± 0.20 ^c^	5.38 ± 0.63 ^b^

Group I: control; group II: CCl_4_; group III: OBE-treated group. Values are mean ± SE (*n* = 5). The presence of different superscripts means significant differences between groups in the same row. ANOVA test followed by Duncanʼs multiple comparisons between groups at *p* < 0.05 were employed.

**Table 4:** Effects of OBE treatment on the antioxidant profile in CCl_4_ fibrotic liver.

Parameter	Group I	Group II	Group III
**SOD (U/ mg protein)**	62.6 ± 2.1 ^c^	18.92 ± 1.08 ^a^	39.6 ± 1.03 ^b^
**CAT (U/ mg protein)**	1.95 ± 0.06 ^c^	0.883 ± 0.03 ^a^	1.3 ± 0.062 ^b^
**GSH (µM/g liver)**	10.6 ± 0.23 ^c^	2.4 ± 0.18 ^a^	6.2 ± 0.31 ^b^
**TAC (µmol/g liver)**	4.4 ± 0.05 ^c^	2.1 ± 0.06 ^a^	3.6 ± 0.02 ^b^

Group I: control; group II: CCl_4_; group III: OBE-treated group. Values are mean ± SE (*n* = 5). The presence of different superscripts means significant differences between groups in the same row. ANOVA test followed by Duncanʼs multiple comparisons between groups at *p* < 0.05 were employed.

### 2.4. OBE Ameliorates CCl_4_-Induced Liver Fibrotic Injury

**Figure 1:**
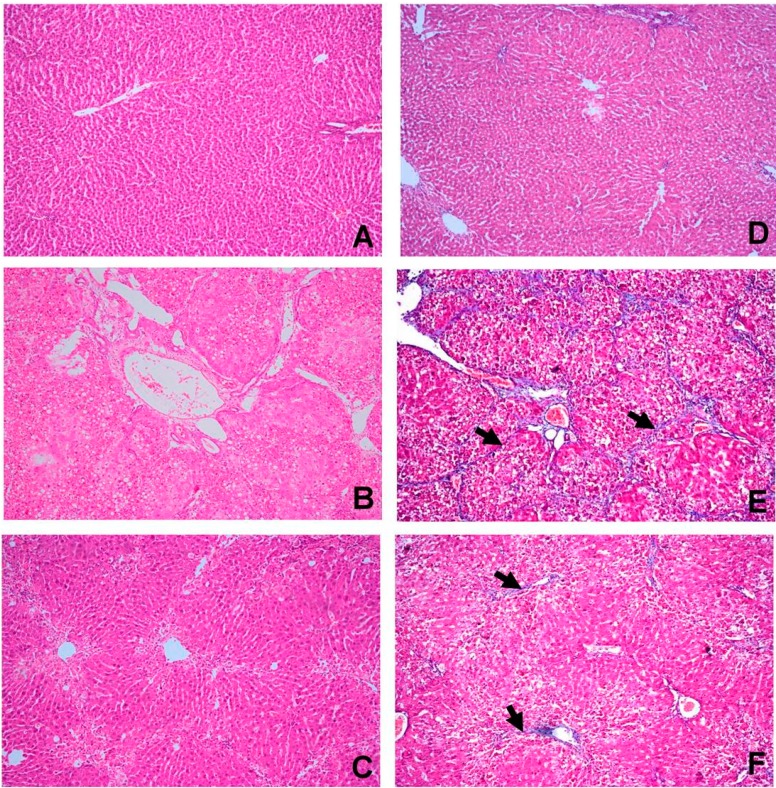
Histopathological examination of livers from different groups. (**A**–**C**) H & E staining; (**D**–**E**) Masson’s trichrome. (**A**) The control group (I) showing normal cellular architecture (H & E ×100); (**B**) CCl_4_ group (II) showing extensive hepatocellular necrosis, marked fatty degeneration, dilation of portal blood vessels and marked fibrous bridging with collagen septa formation (H & E ×100); (**C**) CCl_4_ + OBE group (III) showing moderate centrilobular hepatic necrosis with mild vacuolar degeneration of hepatocytes and thin collagenous septa formation (H & E ×100); (**D**) The control group (I) showing normal collagen fibers in the area of portal and central vein (Masson’s trichrome ×100); (**E**) CCl_4_ group (II) showing extensive collagen deposition (arrow) and pseudolobular formation (Masson’s trichrome ×100); (**F**) CCl_4_ + OBE group (III) showing slight accumulation and spread of collagen fibers (arrow) (Masson’s trichrome ×100).

The control group showed normal hepatic architecture (Figure 1A). The CCl_4_ group revealed severe centrilobular necrosis, marked fatty degeneration of the hepatocytes and abundant collagen fibers in the portal and central areas forming fibrous bridges extending from portal to portal, portal to central and central to central region (Figure 1B). The CCl_4_ + OBE group showed mild centrilobular necrosis, mild to moderate fatty degeneration of the hepatocytes and the collagen fibers were thinner than those observed in CCl_4_ group (Figure 1C).

Masson’s trichrome staining of the liver in different groups was performed to assess collagen fiber distribution. The control group showed normal distribution of collagen (Figure 1D). The CCl_4_ group revealed extensive collagen deposition, bridging fibrosis and pseudolobule formation (Figure 1E). In the CCl_4_ + OBE group, the collagen deposition was reduced (Figure 1F) and pseudolobules were formed by thin collagenous septa. The histopathological grading of liver fibrosis in the different groups is summarized in Table 5.

**Table 5:** Effect of OBE on the pathological grading of CCl_4_-induced liver fibrosis in rats.

Group	*n*	Pathological Grading of Hepatic Fibrosis							*p*-Value
		0	I	II	III	IV	V	VI	
**Control**	6	6	0	0	0	0	0	0	-
**CCl_4_ model**	6	0	0	1	1	3	1	0	0.00 ^a^
**CCl_4_+OBE**	6	0	1	4	1	0	0	0	0.011 ^b^

Results are presented as the mean of ten fields. *n*: number of rats. ^a^: significant difference from control group at *p* < 0.01; ^b^: significant difference from model group at *p* < 0.05.

### 2.5. OBE Inhibits α-SMA Expression in CCl_4_-Induced Fibrosis

**Figure 2:**
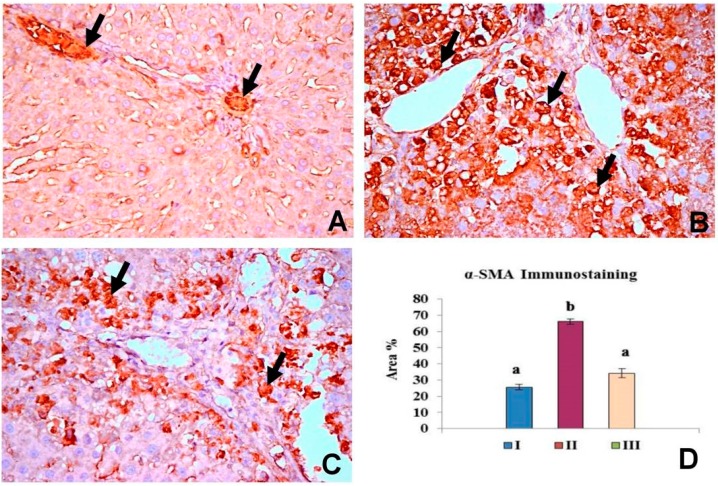
Pattern of immunohistochemistry staining for α-SMA in the liver of different groups. (**A**) The control group (I) showing normal expression of α-SMA positive staining in the smooth musculature of the blood vessels (arrow) (×400); (**B**) CCl_4_ group (II) showing intense immunopostive reaction (arrow) in myofibroblasts cells (×400); (**C**) CCl_4_ + OBE group (III) showing reduction in the areas of immunopostive cells (arrow) (×400); (**D**) Bar chart represents the α-SMA immunopositivity expressed as % area. Mean values with different superscripts are significantly different (*p* < 0.05).

Figure 2 summarized the results of immunohistochemical evaluation of α-SMA expression in the control group (I), CCl_4_ group (II) and CCl_4_ + OBE group (III). α-SMA immunoreactivity was characteristically cytoplasmic and the cytoplasm was stained dark brown color. The control group showed normal expression of α-SMA positive staining about 25.59% area of immunoreactivity which limited to smooth musculature in the blood vessels (Figure 2A). In CCl_4_ group and CCl_4_ + OBE group, α-SMA positive cells were appeared to extend along collagen septa bridging portal areas and central areas (Figure 2B,C). α-SMA expression in the myofibroblasts of CCl_4_ + OBE treated groups (32.37% area) showed significant reduction compered to CCl_4_ group (66.14% area).

### 2.6. OBE Up-Regulates HGF Expression in CCl_4_-Induced Fibrosis

**Figure 3:**
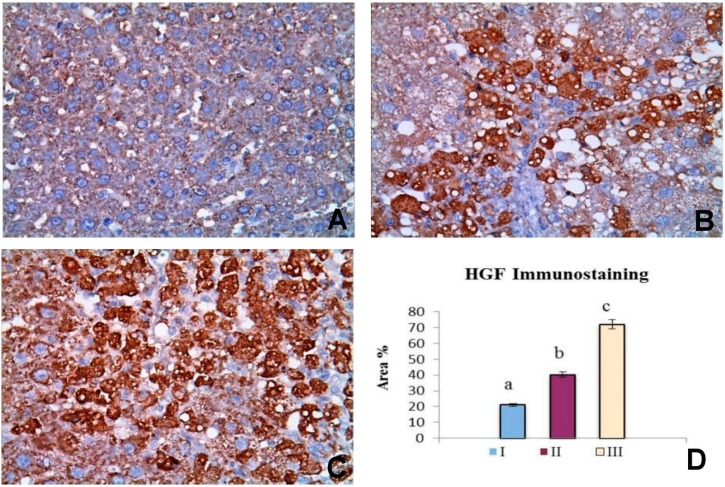
Pattern of immunohistochemistry staining for HGF in the liver of different groups. (**A**) The control group (I) showing weak immunopostive reaction (×400); (**B**) CCl_4_ group (II) showing immunopostive staining in number of cells (×400); (**C**) CCl_4_ + OBE group (III) showing intense immunopostive reaction (×400); (**D**) Bar chart represents the HGF immunopositivity expressed as area %. Mean values with different superscripts are significantly different (*p* < 0.05).

Figure 3 summarized the results of IHC evaluation of HGF expression in the control group (I), CCl_4_ group (II) and CCl_4_ + OBE treated group (III). HGF immunoreactivity was characteristically cytoplasmic and the cytoplasm was stained dark brown color (Figure 3). HGF expression in the hepatocytes of CCl_4_ + OBE treated groups (72.07 area %) was significantly greater than the control group (21.19 area %) and CCl_4_ group (40.28 area %).

**Figure 4:**
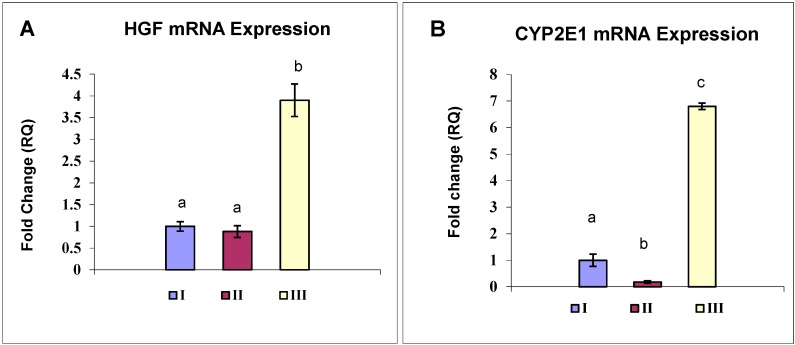
Expression level of HGF (**A**) and CYP2E1 (**B**) genes in the different groups. Group I: vehicle control; group II: CCl_4_; group III: OBE-treated. The steady-state levels of mRNA in the liver were analyzed by real-time PCR assay. Values are presented as means ± SD (*n* = 6). GAPDH was used as an invariant internal control for calculating mRNA-fold changes. Mean values with different letters are significantly different (*p* < 0.05).

On the other hand, HGF mRNA expression level showed no significant change between normal and model group, whereas, OBE-treated rats expressed about 4-fold higher HGF as compared to control (Figure 4A).

### 2.7. OBE Reverses the CYP2E1 Down-regulation Induced in a CCl_4_-Fibrosis Model

Real-time quantitative PCR analysis showed a significantly decreased CYP2E1 mRNA level in CCl_4_-fibrosis group (II) to 18.4% of that of the control rats (I). OBE treatment (III) markedly attenuated CYP2E1down-regulation by increasing its mRNA level to +6.85 folds of that in the normal control (Figure 4B).

### 2.8. Discussion

Hepatic fibrosis is a complex dynamic reaction that is triggered by diverse types of injurious insult, such as viral, alcoholic, drug, or chemical toxicity [3]. The molecular mechanisms underlying fibrosis are basically the same, regardless of its origin. Following chronic liver injury, a series of programmed molecular changes take place. These changes are characterized mainly by activation of hepatic stellate cells (HSCs) into a myofibroblast phenotype which acquires the ability to express and deposit large quantities of collagen [4].

With regards to additional factors mediating the progression of fibrosis, we considered the potential role of oxidative stress and ROS. Oxidative stress represents a common link between the different forms of chronic liver injury and fibrosis. ROS play a crucial role in the initiation of the fibrogenic pathway by integrating various profibrogenic factors that trigger a series of molecular and cellular events implicated in HSCs activation [1]. In this context, an agent which inhibits ROS-mediated fibrogenesis might yield great potential therapeutic benefits for liver fibrosis. We report here that OBE exhibited antifibrotic effects in CCl_4_-fibrotic rats.

Herbal products, supported by their safety, cost-effectiveness and versatility, are enjoying growing worldwide popularity to treat different inflammatory conditions. Many of these plants have been tested for potential hepatoprotective and antifibrotic activity. The antioxidant activity of these products could be the common mechanism by which they exert their protective effects [10].

*Ocimum* is known for its multi-therapeutic potentials [11,12]. Essential oil of basil has been proved to be a potential radical scavenger [12]. It is assumed that its bioactive phytocompounds are responsible for many of the profound medicinal effects of basil [13]. Although much is known about the hepatoprotective effect of *Ocimum* [7,8,14], some aspects of its mechanism of action have yet to be fully understood.

In the present study, OBE showed a unique chemical profile in which the most abundant constituent was the oxygenated monoterpene *iso*-menthone (Table 1). OBE chemical composition (Table 1), compared to previous data, showed good agreement with the results of Hassanpouraghdam *et al.* [15] who reported an OB chemotype in which the oxygenated monoterpenes (menthone, isoneomenthol and menthol) were the predominant components. On the other hand, Ismail [16], Stefan *et al.* [17] and others found linalool as the major constituent in OBE. Nevertheless, the pronounced difference in regard to isomenthone content, high content of 1,8-cineole (13.9%) and methyl eugenol (7.5%) maintained certain level of chemosimilarity with most of the previous reports of OB [16,17,18]. These chemotypic variations may be due to the geographical, climatic, and soil conditions which in turn may affect the chemical composition and other secondary metabolites of the plant [19]. There aren’t direct evidences for the antifibrogeic effect of any of the OBE constituents, but a previous study showed that isomenthone has antiapoptotic action against tumor necrosis factor-α, one of profibrogenic cytokines [20]. Such a mechanism may explain the antifibrogenic effects of OBE observed in the current study. Moreover, 1,8-cineole was found to be an anti-inflammatory and hepatotonic acting by inducing p450 detoxification enzymes. Also, methyl eugenol acts as antioxidant and anti-inflammatory [21]. Triterpenoids of OBE were found to be effective in inhibiting CCl_4_-induced hepatotoxicity in experimental models [9].

In the present investigation, the efficacy and mechanism of OBE to attenuate liver fibrosis were studied using CCl_4_ fibrosis as an experimental model, which is one of the best characterized models that is superficially similar to human cirrhosis [22]. CCl_4_ is metabolized by cytochrome p450 (CYP2E1 isoform) into the hepatotoxic radicals trichloromethyl CCl_3_^•^ and Cl_3_COO^•^ that covalently bind to cell constituents leading to lipid peroxidation and severe hepatic damage with marked elevations of serum AST and ALT [23]. LPO initiates a chain of events: hepatocyte necrosis, activation of the inflammatory cells including macrophages, activation of HSCs, and the release of fibrogenic mediators Thus, the CCl_4_ model resembles all the important characteristics of human liver fibrosis, including inflammation, regeneration, fibre formation and potentially fibrosis regression [24,25].

Since the oxidant/antioxidant imbalance is thought to be a key step in the development of fibrosis, oxidative stress has been carefully investigated in this study. MDA is a prooxidant produced as secondary metabolite of lipid peroxidation and a good indicator of cell membrane impairment [26]. The increased MDA levels in the liver of CCl_4_ group suggests enhanced lipoperoxidative liver damage (Table 3). In the same context, NO, a highly reactive oxidant is extensively generated by CCl_4_-administration due to overexpression of inducible nitric oxide synthase gene (iNOS) by activated HSCs in response to inflammatory cytokines [27]. In the present study, liver NO content significantly increased in CCl_4_-treated rats (Table 3). Those findings were consistent with previous studies that reported a positive correlation between the level of NO and the degree of hepatic fibrosis [28,29]. NO reacts with the superoxide anion to form a potent and versatile oxidant, peroxynitrite, which promotes HSC activation. Such a mechanism could support the contention that NO plays an active role in the progression of liver fibrosis and hepatocellular damage [30].

Under normal circumstances, the body is endowed with effective antioxidant systems to combat the menace of oxidative stress. Though, in extreme oxidative challenges, such as those observed in CCl_4_ hepatotoxicity_,_ the body’s antioxidants machineries are overwhelmed [28]. In the current study, as expected, CCl_4_ caused marked hepatotoxic changes. High ALT, AST and the histopathological finding confirmed CCl4-induced hepatocellular damage (Table 2 and Figure 1). The elevated LPO and NO indices in the CCl_4_ group (Table 3), together with the reduced activities of SOD and CAT and the diminished level of GSH (Table 4) suggested a state of oxidative hepatic damage. Evaluation of TAC verifies the overall oxidative status and provides more accurate information compared to that obtained by the measurement of individual antioxidant parameters [5]. The obtained low levels of TAC in CCl_4_ intoxicated rats reflected the cumulative damaging effects of CCl_4_ against all antioxidants present in the liver (Table 4).

The histopathological analysis provided an initial evidence of the CCl_4_-induced fibrosis in liver. True fibrotic bridging was observed after 8 weeks of continuous treatment, corresponding to approximately 16 injections (Figure 1). Also, other degenerative changes including cytoplasmic vacuolization, fatty degeneration and congestion of blood vessels were observed (Figure 1). Similar findings were obtained by Sakr *et al.* [7] and Yacout *et al.* [14]. However, histopathological examination of the liver is considered the standard method for diagnosis and assessing the hepatic fibrosis and cirrhosis especially with Masson’s trichrome staining. Of note, HSCs with α-smooth muscle actin (α-SMA) expression play a key role in pathophysiological mechanism of hepatic fibrosis. Thus, α-SMA is a reliable unique marker of fibrosis and could be useful in monitoring the efficacy of the antifibrotic therapy [31,32,33]. In this study, immunohistochemical analysis showed an excessive α-SMA expression in CCl_4_-fibrotic livers compared to limited expression in normal livers (Figure 2), confirming that CCl_4_ stimulated the activation of HSCs in the rat model and agreed with [34]. OBE significantly improved the liver histology and resolved the fibrotic changes induced by CCl_4_ and decreased its progression (Figure 1, Table 4), with a marked reduction of in α-SMA immunoreactivity in hepatocytes (Figure 2).

Based on the present results, the ability of OBE to reverse of the severe alterations in the liver injury markers (Table 2) is a clear indication of the improvement of the functional status of hepatocytes with preservation of cellular architecture [7]. Additionally, antioxidant effect of OBE suggests the inhibition of their stimulation of collagen synthesis [35]. The ability of OBE to decrease collagen deposition (Figure 1) α-SMA production (Figure 2) indicates the suppression of HSCs activation, as well [36]. Both mechanisms confirm an antifibrotic activity of OBE. In accordance with these results, Sakr *et al.* [7] reported the protective effect of OB extract against liver damage and fibrosis induced by CCl_4_.

The ability of the liver to regenerate after injury is of a special concern for the maintenance of its important functions in metabolism and xenobiotic detoxification. In a cirrhotic liver, the regenerative ability is impaired. There is much evidence to suggest that liver regeneration in human progresses similarly to rodents and many of the same growth factors and signaling pathways are relevant. This is important to inform clinical knowledge and strategies to bolster regeneration in chronic liver disease [37].

A complex set of interactions of profibrotic and antifibrotic cytokines and secreted proteins contribute to the orchestration of liver regeneration. These proteins include profibrotic proteins as transforming growth factor-β (TGF-β), connective tissue growth factor (CTGF) and platelet-derived growth factor-β (PDGF-β). Much information has recently been obtained concerning the central role of TGF-β and its signal transduction pathways through which the TGF-β action is enhanced by CTGF and fibronectin but suppressed by TNF-α and interferone-γ [37]. Several herbal compounds exert their antifibrotic effects through blocking TGF-β1-mediated fibrogenesis [38].

Promising results have been obtained with the use of HGF either as a recombinant protein or by gene therapy in a wide variety of experimental fibrosis models [39]. HGF is a multifunctional cytokine acting via activation of the tyrosine kinase receptor Met, encoded by the proto-oncogene c-met [37]. HGF elicits mitogenic, motogenic, and morphogenic properties with a great ability to induce liver regeneration and to drive hepatocytes into DNA synthesis [37]. Most of liver-related studies indicated that HGF is a cytoprotective, regenerative molecule as well as an endogenous antifibrotic factor that inhibits the accumulation of ECM and fibrogenesis *in vivo* [40,41].

Several mechanisms have been proposed to explain the beneficial effects of HGF in ameliorating liver fibrosis. HGF accelerates the degradation of ECM by regulating proteinases involved in the breakdown of ECM proteins such as matrix metalloproteinases/tissue inhibitors of metalloproteinases, and plasminogen activators/plasminogen activator inhibitors. HGF also stimulates fibronectin gene expression, a major component of extracellular matrix, suggesting that it may modulate matrix remodeling and, thereby, the restoration of a normal extracellular environment [42]. Additionally, HGF may exert its protective effects via anti-apoptotic and anti-inflammatory signals. Recent data suggest that HGF could limit HSCs proliferation, and collagen formation by down-regulation of fibrotic genes. Interestingly, there is good evidence for HGF antagonizing TGF-β signaling by reducing TGF-β mRNA levels prevent progression of fibrosis [42]. HGF effectively block TGF-β-mediated cellular transdifferentiation into α-SMA-producing cell both *in vivo* and *in vitro* [43]. These effects of HGF on liver may work in concert to limit liver damage and to restore normal liver structure and function. Promising results have been obtained with the use of HGF either as a recombinant protein or by gene therapy in a wide variety of experimental fibrosis models [39]. Recently, HGF-based molecules are available as regenerative or anti-tumor drugs [44]. Few reports have studied the effects of medicinal plants on HGF expression or the significance of HGF in the mechanism of these products [43]. This encouraged our team to study the role of HGF in the hepatoprotective and antifibrogenic activities of OBE.

In the current investigation, a considerable level of HGF protein was detected in fibrotic livers (Figure 3B). However, HGF mRNA level showed no significant change in response to CCl_4_-fibrosis (Figure 4A). These findings agreed with previous studies reported that HGF expression increases at early stage following the onset of liver injury and then decreases greatly later in a manner reciprocal to the progression of fibrosis [43]. We could contribute the elevated immunodetectable HGF level in CCl_4_-treated rats to an extrahepatic HGF endocrine production [38]. In the OBE-treated group, the enhancement of liver HGF mRNA and protein expression (Figure 3 and Figure 4) and the reduction of α-SMA (Figure 2) might have contributed to the reduced hepatic fibrosis in this group. However, the definitive mechanism by which OBE augments HGF expression remains obscure. Future study is needed to provide more complete answers.

In conjunction with the increase in HGF mRNA and protein levels, we analyzed the CYP2E1 mRNA levels as indicator for the liver detoxifying capacity. As expected, CCl_4_ induced a significant reduction of CYP2E1mRNA level (Figure 4B). This specific CYP2E1 dysregulation in CCl_4_ hepatic injury was previously reported by Sakr *et al.* [7] and may result from a direct attack of reactive CCl_4_ metabolites leading to CYP2E1 transcript degradation. Moreover, other mechanisms such as an inhibition of CYP2E1 transcription subsequent to inflammatory responses could also have a role in CYP2E1 mRNA decrease [45]. An interesting observation in our study was the total blockade of CCl_4_-induced CYP2E1 downregulation by OBE treatment (Figure 4B). These data suggest that the hepatoprotective effect of OBE possibly due to prevention of CCl_4_-induced CYP2E1 downregulation. Monitoring of the drug metabolizing system (CYP P450) in fibrotic liver injuries is of critical concern because of its significance in guiding the therapeutic use and dosage adjustment in patient of liver fibrosis [46].

## 3. Experimental Section

### 3.1. Chemicals and Reagents

High grade CCl4 (99.8%) was purchased from BDH (Poole, UK). RNA extraction kit and QuantiFast SYBR Green PCR Kit were obtained from Qiagen (Hilden, Germany). Reverse transcription system was obtained from Thermo Scientific (Meridian Rd, Rockford, IL, USA)*.* Anti-α-SMA and anti-HGF were supplied by Santa Cruz Biotechnology Inc. (Dallas, TX, USA). The chemicals 5,5-dithiobis-2-nitrobenzoic acid (DTNB), dihydrogen phosphate, trichloroacetic acid, thiobarbituric acid and all other chemicals used in the experiment were of analytical grade and purchased from Sigma Chemical Company (St. Louis, MO, USA).

### 3.2. Plant Material

*Ocimum basilicum* leaves were purchased from the local market (Harraz Drug stores, Bab El-Khalk, Cairo, Egypt). Voucher specimen of the plant material was deposited at the Department of Biochemistry, National Organization of Drug Control and Research (NODCAR), Giza, Egypt.

### 3.3. Preparation and Characterization of OBE

Extraction of the essential oil was carried out as described by Ismail [16]. The constituents of OBE were identified using gas-liquid chromatography coupled to mass spectrometry (GC–MS). GC–MS analysis was carried out using a HP-5890 GC instrument equipped with Hp-5872 mass spectrometer (Agilent Technologies, Palo Alto, CA, USA). Helium was used as carrier gas at a flow rate of 1.0 mL/min. Oven temperature program was 70 °C to 300 °C for 10 min for the GC, 270 °C for the injector and 300 °C for the mass detector. Retention indices (RI) were determined using retention times (RT) of authentic compound that was injected after the essential oil under the same chromatographic conditions. The compounds were identified by comparison of retention indices (RI, HP-5) with those reported in the literature and by comparison of their mass spectra with the Wiley GC/MS Library and Mass Finder 2.1 Library. The quantification was done by built-in data-handling program of the equipment. The composition was reported as relative percentage of total peak area.

### 3.4. Animals and Experimental Design

Six week old male albino rats, weighing 150–170 g, were obtained from the animal house of Research Institute of Ophthalmology (Giza, Egypt). Rats were maintained under standard conditions (temperature 25 ± 1 °C, humidity 55% ± 5% and lights on from 06:00 to 18:00 h) with free access to food and water in accordance to the study protocol approved by the ethical committee of the faculty of veterinary medicine, Cairo University, for animal care and experimentation. Rats were equally divided into three groups (ten rats each). Group I (control), was given corn oil (2 mL/kg, IP). Group II (fibrosis model) was given CCl_4_ 1:4 mixture with corn oil (2.5 mL/kg, IP) twice weekly for eight weeks, and group III was given CCl_4_ and OBE (200 mg/kg, IP), daily from the third week.

### 3.5. Sampling

Twenty four h after the last injection, animals were anesthetized with ethyl ether. Blood samples were collected by retro-orbital puncture and centrifuged at 4000 rpm/10 min to separate the serum for measurement of aminotransferases. Thereafter, all the animals were sacrificed, the whole liver tissue were immediately removed, rinsed in ice-cold normal saline and kept at −80 °C until further analyses.

### 3.6. Biochemical Analyses

#### 3.6.1. Hepatotoxicity Evaluation

Liver injury markers, serum alanine aminotransferase (ALT) and aspartate aminotransferase (AST), were estimated according to Reitman and Frankel [47].

#### 3.6.2. Oxidative Stress Biomarkers and Antioxidant Profile

Liver samples were homogenized in 0.1 M cold phosphate buffer saline (pH 7.4) using a Teflon pestle to prepare 10% homogenates. The homogenates were centrifuged at 14,000× *g* for 15 min at 4 °C. The supernatant was used for measurement of liver LPO expressed as malondialdehyde (MDA) [48], nitric oxide concentration (NO) [49], reduced glutathione (GSH) [50], superoxide dismutase (SOD) [51], catalase (CAT) [52], and total antioxidant capacity (TAC) using commercial kits (Biodiagnostic, Cairo, Egypt). Protein concentration in the supernatant was estimated according to Bradford [53] using bovine serum albumin as a standard.

### 3.7. Histopathological Examination

The liver tissues from the different groups were fixed in 10% neutral buffered formalin and routinely processed for paraffin embedding to obtain 4 µm sections. Liver sections were stained with hematoxylin and eosin stain and Masson’s trichrome stain for assessment of fibrosis [54]. A numerical scoring system was employed to assess the grade of fibrosis following the criteria of Ishak system [55], as follow: 0, no fibrosis (normal); 1, fibrous expansion of some portal areas ± short fibrous septa; 2, fibrous expansion of most portal areas ± short fibrous septa; 3, fibrous expansion of most portal areas with occasional portal to portal (P-P) bridging; 4, fibrous expansion of portal areas with marked bridging (portal to portal (P-P) as well as portal to central (P-C); 5, marked bridging (P-P and/or P-C) with occasional nodules (in complete cirrhosis); 6, cirrhosis.

### 3.8. Immunohistochemical Examination

Liver sections were deparaffinized in xylene and rehydrated in graded alcohol. Drops of Hydrogen Peroxide Block (Thermo Scientific) were added to block the endogenous peroxidase activity. The tissues were pretreated with 10 mM citrate buffer, pH 6.0 in microwave oven at 500 W for 10 min for antigenic retrieval. The slides were washed with PBS, and blocked with ultra V Blocking solution (Thermo Scientific) for 5 min. Sections were incubated overnight at 4 °C in a humidified chamber with one of the following primary antibodies: mouse monoclonal antibody to α-SMA diluted 1:100 (Santa Cruz Biotech), and rabbit polyclonal anti-HGF antibody diluted 1:50 (Santa Cruz Biotech). The sections were rinsed again with PBS then incubated with a biotinylated goat anti rabbit and mouse antibody (Thermo Scientific) for 10 min. The sections were rinsed again with PBS. Finally, sections were incubated with Streptavidin peroxidase (Thermo Scientific). To visualize the reaction, slides were incubated for 10 min with 3,3′-diaminobenzidine tetrahydrochloride (DAB, Sigma). The slides were counterstained with haematoxylin then dehydrated and mounted. Primary antibodies were omitted and replaced by PBS for negative controls. The immune-stained sections were analyzed by Leica Qwin 500 Image Analyzer (Leica, Cambridge, UK) in 10 microscopic fields under high-power field (×400) microscope. In each field, the immunopositive (dark brown) area was recorded. Percentage of positive stained area (%) was calculated as mean of 10 fields/slide.

### 3.9. Isolation of Total RNA and Real-Time PCR (qPCR)

Total RNA was purified from 100 mg of liver tissue using Rneasy Mini Kit (Qiagen) following the manufacturer’s protocol. Purity and quantity of the total RNA was measured by using a UV spectrophotometer (Thermo Scientific). The mRNA expression levels of HGF and CYP2E1 genes were assessed using qPCR standardized by co-amplification with the housekeeping gene GAPDH, which served as an internal control. Briefly, the total RNA was reverse transcribed into cDNA by reverse transcriptase kit (Thermo Scientific). cDNA was added to a Quantifast SYBR Green qPCR Master Mix (Qiagen) containing 30 pg/mL of each primer (Table 6).

The thermal profile included 40 cycles of denaturation at 95 °C for 15 s, annealing at 60 °C for 15 s and extension at 72 °C for 45 s. During the first cycle, the 95 °C step was extended to 1 min. The GAPDH gene was amplified in the same reaction to serve as the reference gene. Gene expression levels were calculated and determined following the method described by Livak and Schmittgen [56].

**Table 6:** Primers sequences.

Gene	Primer Sequence		Reference
**HGF**	Forward	GCTTGGCATCCACGATGTTC	[57]
	Reverse	CCCTCACATGGTCCTGATCC	
**CYP2E1**	Forward	TCCAGGTTTGCACCAGACTCT	[58]
	Reverse	TCCTCGCTCCTCCTGAGAAG	
**GAPDH**	Forward	ACCACAGTCCATGCCATCAC	[59]
	Reverse	TCCACCACCCTGTTG CTGTA	

### 3.10. Statistical Analysis

The data were statistically analyzed by the SPSS version 16.0 statistical package. Data were expressed as mean ± standard error (SE). Differences between the groups were assessed using one way analysis of variance (ANOVA). Differences were considered statistically significant at *p* < 0.05 by Duncan’s multiple comparisons.

## 4. Conclusions

In light of all findings, the current study suggest that OBE can evidently inhibit liver fibrosis, which might be related with attenuating oxidative stress, inhibiting α-SMA production, inducing HGF expression and up-regulating CYP2E1 expression. OBE could be a promising complementary treatment to combat liver fibrosis.

## References

[1] Sánchez-Valle V., Chávez-Tapia N.C., Uribe M., Méndez-Sánchez N. (2012). Role of oxidative stress and molecular changes in liver fibrosis: A review. Curr. Med. Chem..

[2] Kim M.D., Kim S.S., Cha H.Y., Jang S.H., Chang D.Y., Kim W., Suh-Kim H., Lee J.H. (2014). Therapeutic effect of hepatocyte growth factor-secreting mesenchymal stem cells in a rat model of liver fibrosis. Exp. Mol. Med..

[3] Liedtke C., Luedde T., Sauerbruch T., Scholten D., Streetz K., Tacke F., Tolba R., Trautwein C., Trebicka J., Weiskirchen R. (2013). Experimental liver fibrosis research: Update on animal models, legal issues and translational aspects. Fibrogenesis Tissue Repair.

[4] Tacke F., Weiskirchen R. (2012). Update on hepatic stellate cells: Pathogenic role in liver fibrosis and novel isolation techniques. Expert Rev. Gastroenterol. Hepatol..

[5] Breikaa R.M., Algandaby M.M., El-Demerdash E., Abdel-Naim A.B. (2013). Multimechanistic Antifibrotic Effect of Biochanin A in Rats: Implications of Proinflammatory and Profibrogenic Mediators. PLoS ONE.

[6] Shirazi M.T., Gholami H., Kavoosi G., Rowshan V., Tafsiry A. (2014). Chemical composition, antioxidant, antimicrobial and cytotoxic activities of *Tagetes minuta* and *Ocimum basilicum* essential oils. Food Sci. Nutr..

[7] Sakr S.A., El-Abd S.F., Osman M., Kandil A.M., Helmy M.S. (2011). Ameliorative Effect of Aqueous Leave Extract of Ocimum basilicum on CCl_4_-Induced Hepatotoxicity and Apoptosis in Albino Rats. J. Am. Sci..

[8] Meera R., Devi P., Kameswari B., Madhumitha B., Merlin N.J. (2009). Antioxidant and hepatoprotective activities of *Ocimum basilicum* Linn. and *Trigonella foenumgraecum* Linn. against H_2_O_2_ and CCl_4_ induced hepatotoxicity in goat liver. Indian J. Exp. Biol..

[9] Marzouk A.M. (2009). Hepatoprotective Triterpenes from Hairy Root Cultures of *Ocimum basilicum* L.. Z. Naturforsch. C.

[10] Duval F., Moreno-Cuevas J.E., González-Garza M.T., Rodríguez-Montalvo C., Cruz-Vega D.E. (2014). Liver Fibrosis and Protection Mechanisms Action of Medicinal Plants Targeting Apoptosis of Hepatocytes and Hepatic Stellate Cells. Adv. Pharmacol. Sci..

[11] Bilal A., Jahan N., Ahmed A., Bilal S.N., Habib S., Hajra S. (2012). Phytochemical and pharmacological studies on *Ocimum basilicum* linn—A review. Int. J. Curr. Res. Rev..

[12] Nahak G., Mishra R.C., Sahu R.K. (2011). Phytochemical investigation and *in vitro* antioxidant evaluation of some Ocimum species. J. Pharm. Res..

[13] Piaru S.P., Mahmud R., Abdul Majid A.M.S., Daoud Z., Nassar M. (2012). Antioxidant and antiangiogenic activities of the essential oils of Myristica fragrans and Morinda citrifolia. Asian Pac. J. Trop. Med..

[14] Yacout G.A., Elguindy N.M., El Azab E.F. (2012). Hepatoprotective effect of basil (*Ocimum basilicum* L.) on CCl_4_-induced liver fibrosis in rats. Afr. J. Biotechnol..

[15] Hassanpouraghdam B.M., Hassani A., Shalamzari S.M. (2010). Menthone and Estragole-rich Essential Oil of Cultivated *Ocimum basilicum* L. from Northwest Iran. Chemija.

[16] Ismail M. (2006). Central properties and chemical composition of *Ocimum basilicum* essential oil. Pharm. Biol..

[17] Stefan M., Zamfirache M.M., Padurariu C., Truta E., Gostin I. (2013). The composition and antibacterial activity of essential oils in three Ocimum species growing in Romania. Cent. Eur. J. Biol..

[18] Abduelrahman A.H.N., Elhussein S.A., Osman N.A., Nour A.H. (2009). Morphological variability and chemical composition of essential oils from nineteen varieties of basil (*Ocimum basilicum* L.) growing in Sudan. Int. J. Chem. Technol..

[19] Joshi R.K. (2014). Chemical composition and antimicrobial activity of the essential oil of *Ocimum basilicum* L. (sweet basil) from Western Ghats of North West Karnataka, India. Anc. Sci. Life.

[20] Jung E., Byun S., Kim S., Kim M., Park D., Lee J. (2012). Isomenthone protects human dermal fibroblasts from TNF-α-induced death possibly by preventing activation of JNK and p38 MAPK. Food Chem. Toxicol..

[21] Duke J.A. (2003). Handbook of Medicinal Herbs.

[22] Tamayo P.R. (1983). Is cirrhosis of the liver experimentally produced by CCl_4_ and adequate model of human cirrhosis?. Hepatology.

[23] Lamireau T., Desmouliere A., Bioulac-Sage P. (2002). Mechanisms of hepatic fibrogenesis. Arch. Pediatr..

[24] Hsu C.L., Hsu C.C., Yen G.C. (2010). Hepatoprotection by freshwater clam extract against CCl4-induced hepatic damage in rats. Am. J. Chin. Med..

[25] Parola M., Marra F., Pinzani M. (2008). Myofibroblast—Like cells and liver fibrogenesis: Emerging concepts in a rapidly moving scenario. Mol. Asp. Med..

[26] Khan M.R., Ahmed D. (2009). Protective effects of *Digera muricata* (L.) Mart. on testis against oxidative stress of carbon tetrachloride in rat. Food Chem. Toxicol..

[27] Iwai S., Karim R., Kitano M., Sukata T., Min W., Morimura K., Wanibuchi H., Seki S., Fukushima S. (2002). Role of oxidative DNA damage caused by carbon tetrachloride-induced liver injury—Enhancement of MeIQ-induced glutathione S-transferase placental form-positive foci in rats. Cancer Lett..

[28] Sagor A.T., Chowdhury M.R.H., Tabassum N., Hossain H., Rahman M., Alam A. (2015). Supplementation of fresh ucche (*Momordica charantia* L. var. muricata Willd) prevented oxidative stress, fibrosis and hepatic damage in CCl_4_ treated rats. BMC Complement. Altern. Med..

[29] Abdel Salam O.M., Sleem A.A., Shafee N. (2010). Effect of trazodone and nefazodone on hepatic injury induced by carbon tetrachloride. Drug Discov. Ther..

[30] Leung T.M., Fung M.L., Liong E.C., Lau T.Y., Nanji A.A., Tipoe G.L. (2011). Role of nitric oxide in the regulation of fibrogenic factors in experimental liver fibrosis in mice. Histol. Histopathol..

[31] Carpino G., Morini S., Ginanni Corradini S., Franchitto A., Merli M., Siciliano M., Gentili F., Onetti Muda A., Berloco P., Rossi M. (2005). Alpha-SMA expression in hepatic stellate cells and quantitative analysis of hepatic fibrosis in cirrhosis and in recurrent chronic hepatitis after liver transplantation. Dig. Liver Dis..

[32] Domitrović R., Jakovac H., Tomac J., Šain I. (2009). Liver fibrosis in mice induced by carbon tetrachloride and its reversion by luteolin. Toxicol. Appl. Pharmacol..

[33] Parikh J.G., Kulkarni A., Johns C. (2014). α-Smooth muscle actin-positive fibroblasts correlate with poor survival in hepatocellular carcinoma. Oncol. Lett..

[34] Rockey D.C., Weymouth N., Shi Z. (2013). Smooth Muscle α Actin (Acta2) and Myofibroblast Function during Hepatic Wound Healing. PLoS ONE.

[35] Parola M., Robino G. (2001). Oxidative stress-related molecules and liver fibrosis. J. Hepatol..

[36] Teraoka R., Shimada T., Aburada M. (2012). The molecular mechanisms of the hepatoprotective effect of gomisin A against oxidative stress and inflammatory response in rats with carbon tetrachloride-induced acute liver injury. Biol. Pharm. Bull..

[37] Kang L.I., Mars W.M., Michalopoulos G.K. (2012). Signals and Cells Involved in Regulating Liver Regeneration. Cells.

[38] Tekkesin N., Taga Y., Sav A., Almaata I., İbrisim D. (2011). Induction of HGF and VEGF in Hepatic Regeneration after Hepatotoxin-Induced Cirrhosis in Mice. Hepato-Gastroenterol..

[39] Zhang J., Zhou S., Zhou Y., Feng F., Wang Q., Zhu X., Ai H., Huang X., Zhang X. (2014). Hepatocyte Growth Factor Gene-Modified Adipose-Derived Mesenchymal Stem Cells Ameliorate Radiation Induced Liver Damage in a Rat Model. PLoS ONE.

[40] Ozawa S., Uchiyama K., Nakamori M., Ueda K., Iwahashi M., Ueno H., Muragaki Y., Ooshima A., Yamaue H. (2006). Combination gene therapy of HGF and truncated type II TGF-beta receptor for rat liver cirrhosis after partial hepatectomy. Surgery.

[41] Gibran A., Masoud M.S. (2012). Bone marrow cells ameliorate liver fibrosis and express albumin after transplantation in CCl_4_-induced fibrotic liver. Saudi J. Gastroenterol..

[42] Xia J., Dai C., Michalopoulos G.K., Liu Y. (2006). Hepatocyte Growth Factor Attenuates Liver Fibrosis Induced by Bile Duct Ligation. Am. J. Pathol..

[43] Zuo C., Xie X., Qiu H., Deng Y., Zhu D., Fan J. (2009). Astragalus mongholicus ameliorates renal fibrosis by modulating HGF and TGF-β in rats with unilateral ureteral obstruction. J. Zhejiang Univ. Sci. B.

[44] Nakamura T., Mizuno S. (2010). The discovery of hepatocyte growth factor (HGF) and its significance for cell biology, life sciences and clinical medicine. Proc. Jpn. Acad. Ser. B Phys. Biol. Sci..

[45] Riddick D.S., Lee C., Bhathena A., Timsit Y.E., Cheng P.Y., Morgan E.T., Prough R.A., Ripp S.L., Miller K.K., Jahan A. (2004). Transcriptional suppression of cytochrome P450 genes by endogenous and exogenous chemicals. Drug Metab. Dispos..

[46] Xie G., Wong C.C., Cheng K.W., Huang L., Constantinides P.P., Rigas B. (2012). Regioselective oxidation of phospho-NSAIDs by human cytochrome P450 and flavin monooxygenase isoforms: Implications for their pharmacokinetic properties and safety. Br. J. Pharmacol..

[47] Reitman S., Frankel S. (1957). A colorimetric method for the determination of serum glutamic oxalacetic and glutamic pyruvic transaminases. Am. J. Clin. Pathol..

[48] Placer Z.A., Crushman L., Johnson B.C. (1966). Estimation of product of lipid peroxidation (malondialdhyde) in biochemical systems. Anal. Biochem..

[49] Miranda K.M., Espey M.G., Wink D.A. (2001). A rapid, simple spectrophotometric method for simultaneous detection of nitrate and nitrite. Nitric Oxide.

[50] Beutler E., Duron O., Kellin B.M. (1963). Improved method for the determination of blood glutathione. J. Lab. Clin. Med..

[51] Nandi A., Chatterjee I.B. (1988). Assay of superoxide dismutase activity in animal tissues. J. Biosci..

[52] Sinha K.A. (1972). Colorimetric assay of catalase. Anal. Biochem..

[53] Bradford M.M. (1976). A rapid and sensitive method for the quantitation of microgram quantities of protein utilizing the principle of protein-dye binding. Anal. Biochem..

[54] Bancroft J.D., Stevens A. (1996). Theory and Practice of Histological Techniques.

[55] Ishak K., Baptista A., Bianchi L., Callea F., de Groote J., Gudat F., Denk H., Desmet V., Korb G., MacSween R.N. (1995). Histological grading and staging of chronic hepatitis. J. Hepatol..

[56] Livak K.J., Schmittgen T.D. (2001). Analysis of relative gene expression data using real-time quantitative PCR and the 2 −ΔΔ CT method. Methods.

[57] Yang L.Q., Li S.J., Cao Y.F., Man X.B., Yu W.F., Wang H.Y., Wu M.C. (2003). Different alterations of cytochrome P450 3A4 isoform and its gene expression in livers of patients with chronic liver diseases. World J. Gastroenterol..

[58] Galal M.K., Khalaf A.A., Ogaly H.A., Ibrahim M.A. (2014). Vitamin E attenuates neurotoxicity induced by deltamethrin in rats. BMC Complement. Altern. Med..

[59] Ogaly H.A., Khalaf A.A., Ibrahim M.A., Galal M.K., Abd-Elsalam R.M. (2015). Influence of green tea extract on oxidative damage and apoptosis induced by deltamethrin in rat brain. Neurotoxicol. Teratol..

